# Isotopic data for bioarchaeological samples from Polynesia (pre-Contact and modern periods)

**DOI:** 10.1016/j.dib.2021.107419

**Published:** 2021-09-28

**Authors:** Chris Stantis

**Affiliations:** Department of Anthropology, National Museum of Natural History, Smithsonian Institution, United States

**Keywords:** Bioarchaeology, Stable isotopes, Pacifica, Island archaeology, Archaeological chemistry

## Abstract

Two datasets are presented in this paper, one from archaeologically-derived human remains and one from modern plants and animals. First, isotopic data (δ^13^C, δ^15^N, δ^34^S, and ^87^Sr/^86^Sr) along with some radiocarbon dates from archaeologically-derived bone and teeth from individuals buried in pre-Contact Tonga and Fiji are provided. The second dataset is comprised of modern plant and animal samples (*n* = 27) collected from the island of Atiu (Cook Islands). δ^13^C and δ^15^N data from these samples are presented, and this data is integrated with other datasets to provide an aggregated tropical Pacific dietary baseline for paleodietary studies in this region. First collected to explore the interactions between diet, mobility, social status, and the physical environment, the archaeologically-derived samples are from two Polynesian islands. From the Tongan island of Tongatapu, two burial mounds designated To-At-1 and To-At-2 and collectively called ‘Atele, are radiocarbon dated to the Complex Centralized Chiefdom period (c. 500—150 BP). The second collection is from the coastal site of Bourewa in the Republic of Fiji, dated to the Vuda Phase (c. 750—150 BP). From these human remains the following tissues and isotopes were analyzed: bone and dentine collagen analysis of δ^15^N and δ^13^C (*n* = 81) as well as δ^34^S where collagen yield was large enough (*n* = 37), tooth enamel ^87^Sr/^86^Sr (*n* = 47), and radiocarbon dates to supplement the relative dating of the burial sites (*n* = 16). The human data provides a relatively large dataset (for the region) to compare to other contemporaneous sites to consider intra- and inter-island dietary practices and consider movement in the wider region (while being mindful of issues with interpretation). The amalgamated dietary baseline has utility for placing archaeological and ecological samples within the tropic Pacific foodweb.

## Specifications Table

**Table:** 

Subject	Social Sciences- Archaeology
Specific subject area	Bioarchaeology
Type of data	Table Figure
How data were acquired	Mass spectrometry instrumentation For carbon and nitrogen isotope analysis: Flash EA 2112 coupled to a DeltaXP continuous-flow isotope-ratio-monitoring mass spectrometer. For sulphur stable isotope composition of human samples: HekaTech EuroVector elemental analyzer coupled to a Delta V Plus mass spectrometer Strontium analysis: Thermo Fisher Neptune plasma ionization multicollector mass spectrometer (PIMMS)
Data format	Raw
Parameters for data collection	For the first dataset, all archaeologically-derived individuals available were sampled. Bone and teeth affected pathologically were avoided. Care was taken to avoid double-sampling the same individual in instances of multiple/commingled burials. For the second dataset, wild plants and animals that might have been consumed by past peoples were selected. No imported fertilizer was used on Atiu, so avoiding this was not a concern. Animals that might have scavenged imported food from middens (e.g., rats, dogs, pigs) were not sampled.
Description of data collection	Bone and dentine collagen analysis of δ^15^N and δ^13^C (and δ^34^S where collagen yield was sufficient) were conducted on 81 samples. In 47 Individuals, tooth enamel was collected for ^87^Sr/^86^Sr analysis. To supplement the dietary and mobility information, 16 bones were analyzed for radiocarbon dating. Modern plant and animals were cleaned, defatted, and analyzed for δ^13^C and δ^15^N values.
Data source location	Institution: University of Otago City/Town/Region: Dunedin Country: New Zealand Latitude and longitude (and GPS coordinates, if possible) for collected samples/data: Note: Latitude and Longitude are given using WGS 84 `Atele, Tongatapu, Tonga: −21.182823, −175.226917 Bourewa, Viti Levu, Fiji: −18.089810, 177.304114 Atiu Island, Cook Islands: −19.9941, −158.108
Data accessibility	Available at the public repository IsoArcH. Repository name: Isoarch https://isoarch.eu/ Data identification number: https://doi.org/10.48530/isoarch.2021.004 Direct URL to data: https://doi.org/10.48530/isoarch.2021.004 See: Salesse, K., Fernandes, R., de Rochefort, X., Brůžek, J., Castex, D., & Dufour, É. (2018). IsoArcH.eu: An open-access and collaborative isotope database for bioarchaeological samples from the Graeco-Roman world and its margins. Journal of Archaeological Science: Reports, 19(June), 1050-1055. https://doi.org/10.1016/j.jasrep.2017.07.030

## Value of the Data

• Archaeologists and ecologists are the main researchers benefiting from these datasets.
• This data can be compared to other archaeological sites to consider inter-island differences in diet and subsistence practices, as well as provide comparative ^87^Sr/^86^Sr data for paleomobility studies. The flora and fauna data (both raw, and secondary aggregated) have utility for building a tropical Pacific isotopic foodweb.
• When interpreting paleodiet, it is important to establish a dietary baseline specific to the region of interest. There are slight but significant variations around the globe for carbon and nitrogen stable isotope values of plant and animal life due to variable rainfall, soil conditions, climate, and other factors [1,2]. The dietary baseline is useful to archaeological paleodiet research where a local baseline could not be collected for placing humans or domesticates within their local foodweb.
• Ecologists will find the modern plant and animal isotopic data useful for placing other organisms within the food web.

## 1 Data Description

**Fig. 1:**
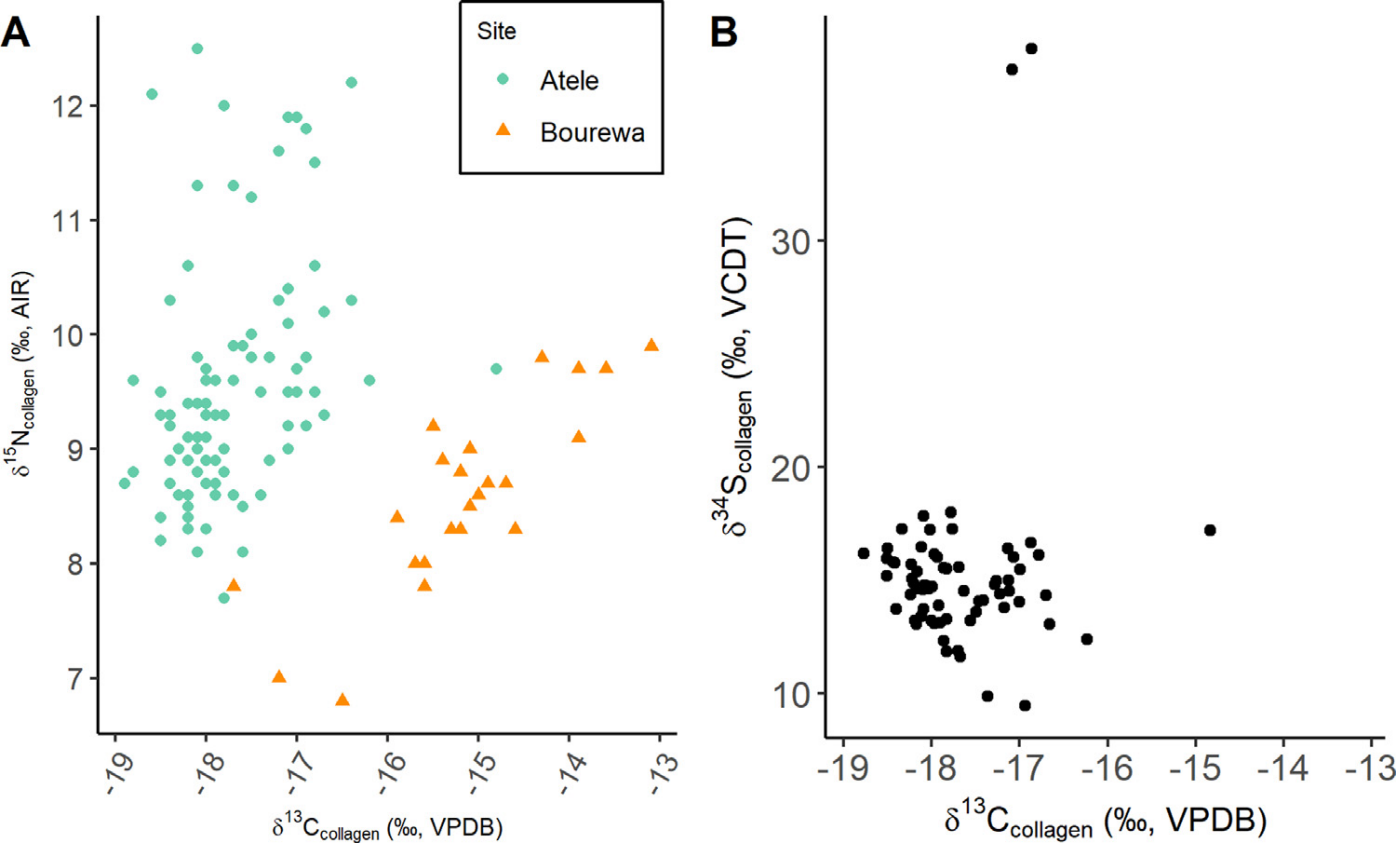
(A) δ^13^C and δ^15^N values from the sites of ‘Atele and Bourewa. (B) δ^34^S and δ^13^C values from the site of ‘Atele.

**Fig. 2:**
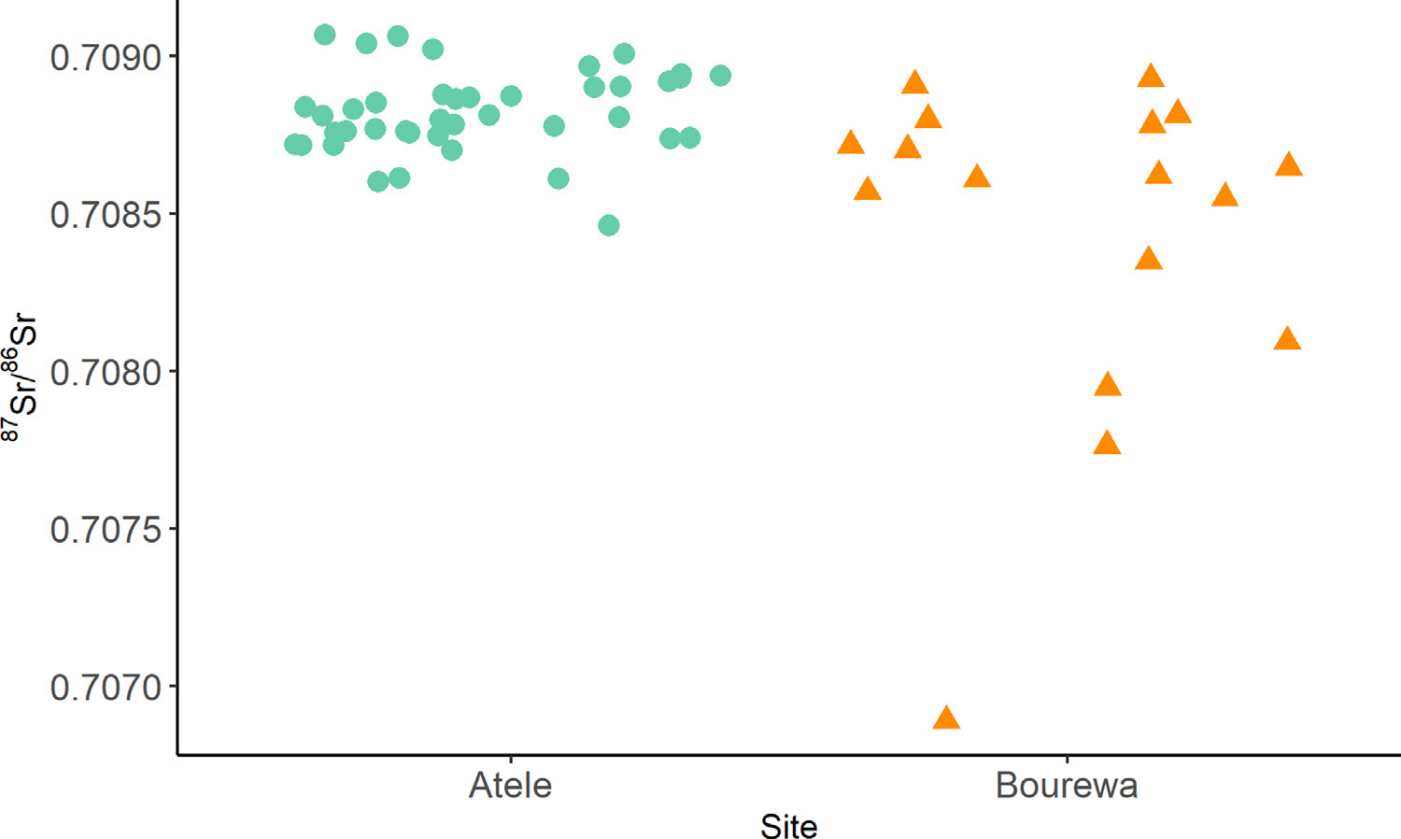
^87^Sr/^86^Sr values from the sites of ‘Atele and Bourewa.

**Fig. 3:**
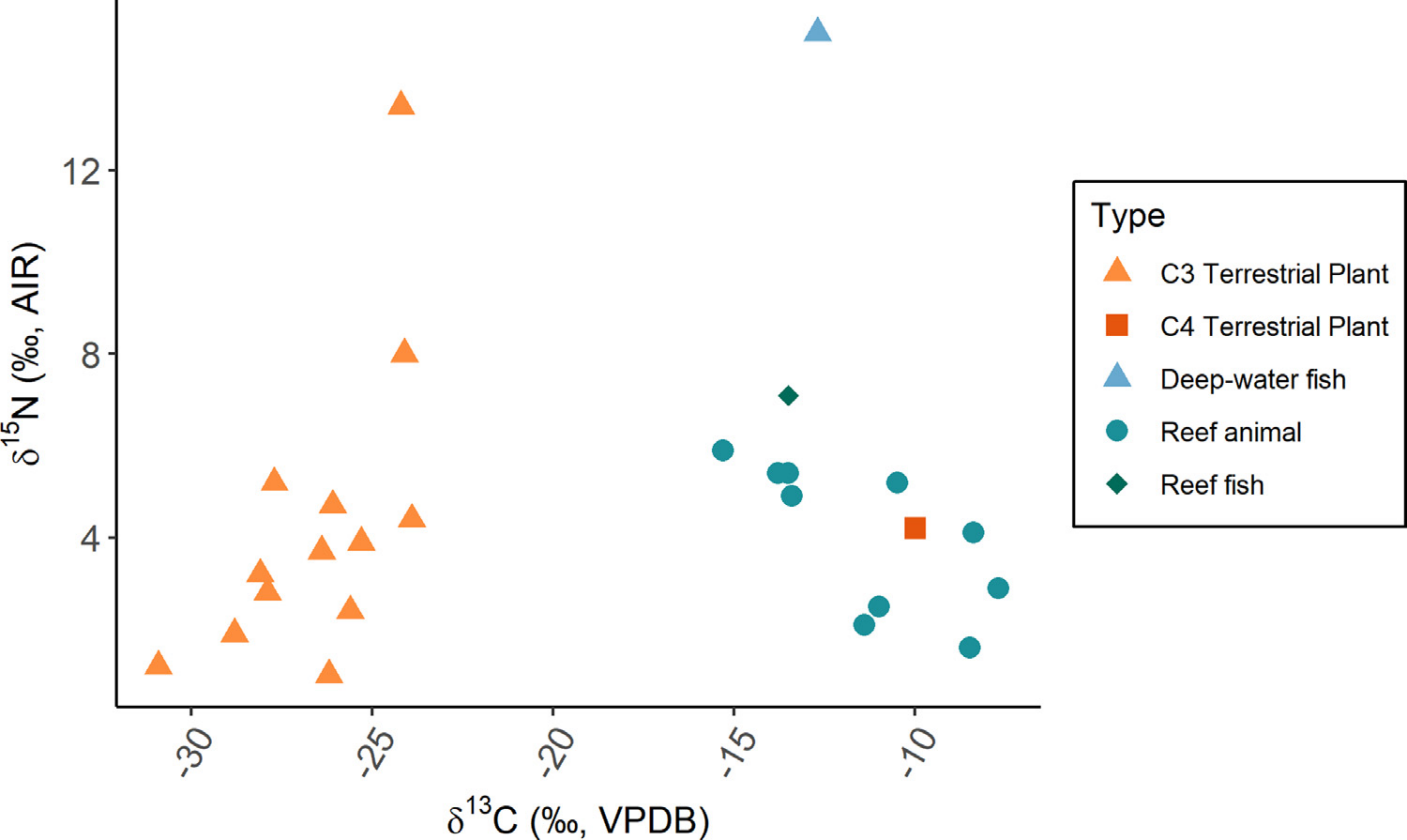
δ^13^C and δ^15^N values from modern plants and animals collected from the Cook Islands. Data corrected for Suess effect (see methods description).

This collection is comprised of multiple isotopic datasets. Firstly, bone and dentine collagen δ^13^C and δ^15^N data from the archaeological sites of ‘Atele and Bourewa (*n* = 81), as well as δ^34^S from ‘Atele where collagen yield was sufficient (*n* = 37) (Fig. 1) in addition to ^87^Sr/^86^Sr from tooth enamel from these sites (*n* = 51) (Fig. 2). δ^13^C and δ^15^N values of 27 modern plants and animals collected from the island of Atiu in the Cook Islands are also provided (Fig. 3). These data are summarized on Table 1. Radiocarbon dates ran to supplement the relative dating at the sites are also provided in the repository. These areas are mapped at the public repository website (see **Data Accessibility** for URL).

**Table 1:** Statistical summary of human data.

		δ^13^C	δ^15^N	δ^34^S		^87^Sr/^86^Sr	
Site	*n*	Mean ± 1 SD	Mean ± 1 SD	*n*	Mean ± 1 SD	*n*	Mean ± 1 SD
‘Atele	41	−17.6 ± 0.8	9.3 ± 0.5	36	14.5 ± 1.7	30	0.7086 ± 0.0001
Bourewa	40	−15.6 ± 1.2	8.8 ± 0.8	0		21	0.7085 ± 0.0005

The archaeological site of ‘Atele (Tongatapu, Kingdom of Tonga) consists of two burial mounds, To-At-1 and To-At-2. These two burial mounds, dated to the Complex Centralized Chiefdom period (c. 500—150 BP), were hypothesized to inter different classes of people in the stratified Tonga culture: commoners and elites, or perhaps commoners and higher-ranking commoners such as chiefly retainers or skilled workers [3]. The site of Bourewa (Viti Levu, Fiji) is roughly contemporaneous with the ‘Atele site being dated to the Vuda Phase of Fijian prehistory (900–150 BP). It is a smaller cemetery than ‘Atele and seems to represent a small community on the coastal fringes of the island, surviving with little inland input, whether terrestrial foodstuffs or inland contact/migration [4].

Fieldwork on the Cook Islands allowed the collection of modern samples of plants and animals that would have been consumed by Pacific peoples pre-European Contact (many of which are still regularly consumed today). These represent both wild plants and marine resources, as well as some plants which were imported by Polynesians and their Lapita ancestors to build a ‘transported landscape’ for settling in new island ecosystems. The statistical summary (Table 2) groups by a general typology; note that reef fish is differentiated from reef “animal” to seperate the fish from the lower trophic level animals it might have been feeding on, e.g., sea urchins and mollusks.

**Table 2:** Statistical summary of baseline plant and animal data from the Cook Islands. Correction for Suess effect included, but no other changes are made to the values (e.g., bone-to-flesh).

		δ^13^C	δ^15^N
Group	*n*	Mean ± 1SD	Mean ± 1SD
C_3_ Terrestrial Plants	13	−26.6 ± 2.1	4.29 ± 3.3
C_4_ Terrestrial Plants	1	−10	4.2
Deep-water Fish	1	−12.7	15
Reef Animal	10	−11.4 ± 2.6	4 ± 1.6
Reef Fish	1	−13.5	7.1

## 2 Experimental Design, Materials and Methods

Methods for bone and dentine bulk collagen analysis and ^87^Sr/^86^Sr analysis of tooth enamel have been described previously [3,4] but are explained in detail here.

### 2.1 Bulk collagen analysis of δ^13^C, δ^15^N, and δ^34^S

Cortical bone fragments approximately 1200–1400 mg were sampled for bulk collagen from bone. Bones displaying pathological changes were excluded from sampling on the principle that changes in the metabolic pathways of the tissue because of disease may affect the isotopic values. For tooth sampling, permanent second molars (maxillary or mandibular, any side) were preferentially chosen as the primary sample over any other tooth type. If second molars were not available, permanent premolars (first or second) were chosen, followed by first molars. For first molars, the root half (R_1/2_) with the apex (A) was used for isotopic analysis; for the premolars and second molar the R_1/2_ closer to the cement-enamel junction (CEJ) was sampled. This created a roughly equivalent time span captured in each root, between ∼5 and 10 years of age.

After sandblasting to remove surface impurities, collagen extraction and purification were carried out using a modified Longin method [5,6] at the Department of Anatomy, University of Otago (New Zealand). Demineralisation and the removal of carbonates, phosphates, and fulvic acids from the samples occur by soaking samples in 15 mL of 0.5 M HCl at 4 °C under an aluminium foil “tent” (to prevent contamination). Every 48 h, the solution was refreshed until a sample was demineralized and was in a pseudomorph form. As samples demineralized at different rates, pseudomorphs were stored in distilled H_2_O at 4 °C until a batch was ready for the next step. Samples were washed three times in distilled water. In order to solubilize the samples and prevent oxidation, samples were gelatinized in ∼8 mL 3.00 pH HCl solution at 70 °C for 48 h. Next, the samples were filtered using a 5-8 μm Elkay Ezee mesh filter (discarding the solids).

Then, they were ultrafiltered using Amicon Ultra-0.5 Centrifugal Filter Units with Ultracel-30 membranes to remove peptides smaller than 30 kDa NMWL; these peptides of lower molecular weight are likely humic contaminants. The ultrafilters are first cleaned to remove exogenous carbon (in the form of glycerine in the ultrafiltration membranes) by centrifuging distilled water two times in the centrifuge at 2300. The goal is to have material remaining between 500 and 1000 µL; centrifuge in 4 min intervals at 3000 rcf, checking between intervals until the goal volume is acquired. The liquid at the bottom of the filter is discarded and the material remaining above the filter was pipetted into labelled microcentrifuge tubes. The samples were frozen and then lyophilized for 48 h.

Stable isotope measurements were conducted at the Department of Human Evolution, Max Planck Institute of Evolutionary Anthropology (Germany). Carbon and nitrogen isotope values were measured simultaneously using a Flash EA 2112 coupled to a DeltaXP continuous-flow isotope-ratio-monitoring mass spectrometer. Sulphur isotope composition was measured by combusting the samples in SO and SO_2_ gas in a HekaTech EuroVector elemental analyzer coupled to a Delta V Plus mass spectrometer. Repeated measurements of working standards EVA-0009 (methionine), SRM 1577b (bovine liver), IAEA-N-1 and -N-2 (ammonium sulphate), IAEA-CH-6 (sucrose), and IAEA-CH-7 (polyethylene) were interspersed throughout the archaeological samples to correct the carbon and nitrogen isotope data. For sulphur, IAEA-S-1 (silver sulphide), IAEA NBS-127 (barium sulphide), IAEA-SO-5 (barium sulphide), SRM 1577b and IVA-001 (casein protein) were interspersed.

Collagen integrity is reported: %C by weight, %N by weight, and C/N ratios as well as %S by weight, C/S and N/S ratios where sulphur was also analyzed. For ancient human remains, collagen samples displaying C/N ratios between 2.9–3.6, %C values between 15–47%, and %N values between 5–17% are considered well preserved [7,8]. For sulphur analysis, samples displaying a C:S ratio of 600 ± 300, N:S ratio of 200 ± 100, and a %S by weight of 0.15–0.35% are considered well-preserved [9]. In this database, all samples are tabulated, including those that do not meet collagen quality criteria.

All dietary stable isotope ratios are reported in delta (δ) notation ([R_sample_/R_standard_]-1) × 1000, where R is the ^13^C/^12^C, ^15^N/^14^N, or ^34^S/^32^S ratio, standardized to the international references standards for carbon (Vienna-Pee Dee Belemnite [VPDB]), nitrogen (Atmospheric Nitrogen [AIR], and sulphur (Vienna-Canyon Diablo Troilite [VCDT]). These values are presented in per mill (‰) notation, or parts per thousand.

### 2.2 Strontium analysis

Enamel samples were prepared and analyzed for strontium isotope ratios at the Department of Human Evolution, Max Planck Institute of Evolutionary Anthropology (Leipzig, Germany) using method outlined by Deniel and Pin [10]. The crown of each tooth (premolars or molar) was sandblasted to remove the outer layer. A small piece of enamel was cut from the tooth using a dental rotary tool. Any dentine attached to the sample was ablated with a diamond-tipped engraving cutter. After being sonicated with 1 mL acetone and rinsed three times with water, samples were purified using an ion exchange method.

In this method, samples are transported to a clean room and placed in lidded 5 ml Teflon® beakers. In each beaker, 2 mL 65% HNO_3_ was pipetted to dissolve the enamel, with dissolution facilitated by a 120 °C hotplate for 2 h. The lids were removed to allow evaporation and the beakers placed back on the hotplate for 10 h.

Evaporation of the solution should leave a small, white pellet in the beaker. 1 mL 3M HNO_3_ were added to each beaker, and then the lidded beakers placed back on the hotplate for another hour to dissolve the pellet. The solution was pipetted to a 2 mL microcentrifuge tube. Extraction chromatographic Eichrom® resin, suspended in ultrapure water, was added to 2 mL columns with frits to create a resin filter ∼0.5 cm thick.

The resin was cleaned with ultrapure water twice, conditioned with 3 mL 3M HNO_3_, and then the sample solution was pipetted into the column. The solution was reloaded 3x to allow optimum strontium extraction. The column was then washed 3x with 3M HNO_3_. Next, 1.5 mL ultrapure water rinses the strontium into a labelled beaker. The elute is evaporated in this uncovered beaker on a 120 °C hotplate for 8 h. The strontium is finally resolved with 2M 3% HNO_3_ on the 120 °C hotplate for 1 h.

The samples, SRM 1486 (bone meal), and blanks were analyzed using a Thermo Fisher Neptune plasma ionization multicollector mass spectrometer (PIMMS). Repeated measurement of international standard SRM 987 (bone meal) was used to ensure accuracy of data, and samples were adjusted using the published value of SRM 987, 0.710240. Isotope dilution analysis was used to obtain the strontium concentration (reported in ppm). A concentration calibration line was created by running the internal standards in each batch at three concentrations, 100 ppb, 400 ppb, and 700 ppb. Signal interference was corrected by measuring ^87^Rb, ^83^Kr, and ^82^Kr. Instrumental mass bias was normalized by measuring for ^88^Sr and using the natural ^88^/^86^Sr ratio of 8.375209.

### 2.3 Radiocarbon dating

All radiocarbon dating of cortical bone from individuals was contracted through the University of Waikato Radiocarbon Dating Laboratory (New Zealand). 0.5—1 g of cortical long bone samples unaffected by pathology were isolated, labelled, and shipped to the contract lab. This laboratory provided the date corrections for marine/lagoon reservoirs and calibrated using the IntCal13 and Marine13 curves in OxCal v.4.2.3 [11].

### 2.4 Modern plant and animal data from the Cook Islands

The edible portion of plants that would have likely been consumed by prehistoric peoples were collected. Plants that may have been fertilized with commercial fertilizers were avoided. All terrestrial plants were collected with the permission of the landowners.

Fish bones were collected from local fishermen. No samples were collected from endangered species. Modern domesticated fauna such as pigs (*Sus scrofa*), chickens (*Gallus gallus domesticus*) and dogs (*Canis lupis familiaris*) were not sampled, as they are fed or scavenge on imported foods. Rats (*Rattus exulans* or *Rattus rattus*) were also excluded as they may have scavenged on imported food. In addition, the use of domesticates from other islands or time periods would have be interpreted with caution as different husbandry practices can create vastly different diets for these animals [12,13].

The plant and animal samples from the Cook Islands were prepared at the University of Otago Department of Anatomy following a de-fatting protocol [14]. Bones were rinsed with distilled water, soaked in a methanol/chloroform solution (2:1 by volume) and sonicated for 3 h. The solution was changed five times during the ultrasonic bath. The bones were then rinsed with distilled water and demineralized following a modified Longin [5] protocol as described in [3].

Animal flesh and plant samples were rinsed in distilled water and placed in the methanol/chloroform solution in polypropylene centrifuge tubes. Samples were rotated and solvent was replaced every 24 h until the solution was clear (approximately five days). The samples were then rinsed again in distilled water, placed in microcentrifuge tubes, and left to dry. Finally, all baseline samples were lyophilized. The freeze-dried samples were sent to Iso-Analytical Limited (United Kingdom) for carbon and nitrogen stable isotope analysis.

The modern plant and animal data provided has been corrected for Suess effect, the change in δ^13^C values by the introduction of large amounts of fossil fuels in the Earth's carbon cycle [15,16]. To enable comparison to archaeological data collections, terrestrial organisms were corrected by +1.5‰. Marine organisms were corrected by +0.86‰ as the oceanic carbon reservoir is more resistant to change [17,18].

## Ethics Statement

*Regarding human samples:*

No permits were required for the described study of the `Atele and Bourewa human remains, which complied with all relevant regulations. These collections are currently curated by the University of Otago Department of Anatomy (Dunedin, New Zealand).

*Regarding Cook Islands modern plant and animal samples:*

Biosecurity export clearance was granted by the Cook Islands Ministry of Agriculture (Biosecurity Service). Import into New Zealand was granted using the University of Otago's Anthropology and Archaeology Departmental import permit. The samples were inspected and granted clearance by the Ministry of Agriculture and Forestry and were transported immediately to the Anthropology and Archaeology transitional facility upon arrival to Dunedin.

## CRediT authorship contribution statement

**Chris Stantis:** Conceptualization, Methodology, Data curation, Writing – original draft, Writing – review & editing.

## Declaration of Competing Interest

The author declares that they have no known competing financial interests or personal relationships which have or could be perceived to have influenced the work reported in this article.
